# Microzoa

**Published:** 1889-07

**Authors:** Samuel Swan

**Affiliations:** New York


### Microzoa.

The vital force, or nature, as it is generally termed, is always endeavoring to expel from the body all poisons of whatever kind, whether generated from without or within the body.

In doing so she makes use of all the forces at her command, and they are numerous and of great variety. Among these are what are called *microzoa*, which means microscopic animal forms. These are innumerable and when the body is in perfect health they are undiscoverable by the most powerful microscopes.

There are innumerable families and a few of them are named bacteria, bacilli, vitriones, microbes, etc. Each family are affected by the particular poison for which they have an affinity, and when a poison invades the system, that family of microbes whose business it is to resist that particular poison, goes vigorously to work to absorb it, and they grow fat on it, and thereby come into the field of vision, and the observer seeing the especial form, says: This is a yellow fever microbe, or a typhoid, or diphtheritic, or some other. Pasteur recognized this, and he takes the microbe of rabies and potentizes it by cultivation, and inserts it into the system by inoculation, and thereby cures the patient.

Why does he cultivate it? Because homœopathy has shown that the *same* poison will not antidote the poison, until by potentization, it becomes a *like*, and according to the homœopathic law, a *like* cures a *like*. Thus the high potency of the rabies poison will cure while the crude poison will not. Because each particular disease showed the particular microbe whose duty it was to attend to that poison, it has been supposed that microbes were the cause of disease, while they are not, or even the result, but are only visible because fattened by the poison.

The firemen of a city are not visible while in their houses, but give the alarm and they swarm to the fire to extinguish it. But they were not the cause of the fire, but because of the fire they became visible. Thus all such inventions as "microbe killers" are useless, for the microbe will not disappear as long as the poison is in the system, while as soon as the poison is antidoted or expelled, the hard-worked microbe grows small till at last invisible to the microscope, but he is there all the same.

Experience has shown that the higher the potency is carried the more rapid and permanent is the result.

Samuel Swan, M.D.

13 West 38th St., New York.

Droll Story Told by a Good Old Doctor.—Dr. Levi Ives is an old practitioner and still in the harness. Once he was summoned to consult with a country physician who had studied medicine under him. The course of treatment was described to Dr. Ives and he found fault with some remedy. "Why, Doctor," remonstrated the other, "when I was studying with you, you gave that remedy in every case." "My son," quoth Dr. Ives, "you remind me of my horse. Once when I was driving along a country road I came to a pool of water at which my horse shied, and I had some difficulty in getting him to pass. The next summer I drove over the same road. When I came to the place where the pool had been there was no pool to be seen, but the horse remembered the place and shied again. Now that shows that the horse had a good memory, but mighty poor judgment."—*New Haven Pal.*

